# A Multichannel Fluorescent Tongue for Amyloid-*β* Aggregates Detection

**DOI:** 10.3390/ijms232314562

**Published:** 2022-11-23

**Authors:** Fei Li, Lingjia Zhou, Xu Gao, Weiwei Ni, Jiabao Hu, Meicen Wu, Shouwang Chen, Jinsong Han, Jin Wu

**Affiliations:** 1 State Key Laboratory of Natural Medicines, National R&D Center for Chinese Herbal Medicine Processing, Department of Food Quality and Safety, College of Engineering, China Pharmaceutical University, Nanjing 210009, China; 2 Department of Neurology, The Second Affiliated Hospital of Nanjing Medical University, Nanjing 210011, China

**Keywords:** Alzheimer’s disease, multichannel, sensor array, amyloid-β protein, machine learning algorithm

## Abstract

Attention has been paid to the early diagnosis of Alzheimer’s disease, due to the maximum benefit acquired from the early-stage intervention and treatment. However, the sensing techniques primarily depended upon for neuroimaging and immunological assays for the detection of AD biomarkers are expensive, time-consuming and instrument dependent. Here, we developed a multichannel fluorescent tongue consisting of four fluorescent dyes and GO through electrostatic and π–π interaction. The array distinguished multiple aggregation states of 1 µM A*β*40/A*β*42 with 100% prediction accuracy via 10-channel signal outputs, illustrating the rationality of the array design. Screening vital sensor elements for the simplified sensor array and the optimization of sensing system was achieved by machine learning algorithms. Moreover, our sensing tongue was able to detect the aggregation states of A*β*40/A*β*42 in serum, demonstrating the great potential of multichannel array in diagnosing the Alzheimer’s diseases.

## 1. Introduction

Alzheimer’s disease (AD) is the most common neurodegenerative disease with complex pathophysiology, accompanied by irreversible memory loss and severe cognitive dysfunction [1]. For AD patients, early diagnosis and monitoring are of great significance. As one of the most vital biomarkers in the diagnosis of AD, amyloid-*β* (A*β*) proteins containing 39–43 amino acid residues exhibit differential aggregation tendencies, with varying aggregation states showing diverse neurotoxicity [2,3,4]. Among them, A*β*40 and A*β*42 are typical species with minor differences that reflected on extra isoleucine and an alanine at its C-terminus of A*β*42 sequence. At present, the detection methods for A*β* aggregates are mainly reliant on the combination of neuroimaging and immunological detection; however, the wide applications of current detecting techniques are greatly limited by high cost, high radiation, time-consuming, complicated operations, and low sensitivity [5,6,7,8,9]. Furthermore, detecting individual biomarkers such as A*β*40 or A*β*42 is powerless in diagnosing AD as a single indicator cannot reflect the progress of AD pathology. Therefore, it is highly beneficial to develop a simple and rapid approach for sensing A*β*40/A*β*42 and their aggregates simultaneously [10,11,12].

Differently from specific probe based on the “lock-key” mechanism, cross-reactive sensor array (or chemical tongue) provides a parallelly sensing strategy for multiple analysts through the pattern responses of sensor array towards the analyst [13,14,15,16,17,18]. In recent years, efforts have been devoted to the design of sensor arrays in detecting bacteria, proteins, environmental pollutants, food security, etc. [19,20,21,22,23,24,25,26,27]. Recently, we have validated that the fluorescent sensor array consisting of electrostatic complexes formed from poly(*para*-phenylene ethynylene)s (PPEs) and graphene oxide (GO) is capable of successfully distinguishing various A*β* aggregates [28]. To further enhance the sensitivity, we intend to introduce a wider range of A*β* aggregates-specific and non-specific recognition moieties into the sensing system [29]. Meanwhile, in another recent work, we demonstrated that a single-well multichannel sensor array can greatly improve detection efficiency and increase the sensitivity of cross-responses; hence, the strategy was employed in this work [30].

In this study, a multichannel sensor array was constructed with one five-element complex by using four dyes with different emission wavelengths and GO for the parallel discrimination of different aggregation states of A*β*40/A*β*42 in a single measurement. In our smart sensing system, peptide nuances and different aggregation states can generate various dissociation or adsorption between A*β*40/A*β*42 proteins and sensor elements, leading to multiple fluorescent responses in a single measurement. The array has achieved the discrimination of A*β*40/42 aggregates with 100% prediction accuracy in PBS through 10-channel signal outputs. Meanwhile, a simplified 6-channel sensor array, optimized by machine learning algorithm, was also able to generate excellent discrimination. Additionally, the anti-interference detection results were also satisfactory. Overall, our multichannel sensor array has revealed powerful ability in discriminating A*β*40/42 aggregate species, indicating the potential of multichannel array in disease diagnosis.

## 2. Results and Discussion

**Figure 1:**
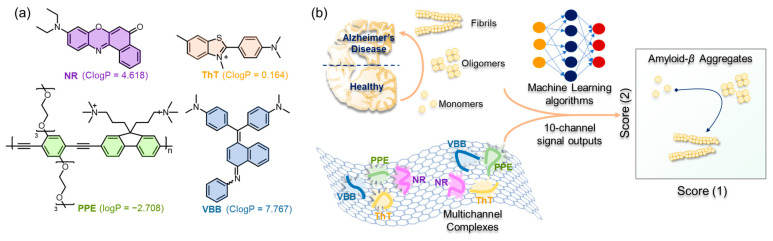
(**a**) Structure and ClogP (predicted by ChemBioDraw Ultra 18.0) of PPE, ThT, NR and VBB. (**b**) Schematic diagram of a multichannel sensor array for recognition of A*β*40/A*β*42 aggregates.

In this study, cationic PPE [28] and three commercially available dyes (Thioflavin T (ThT), Nile Red (NR) and Victoria Blue B (VBB)) were combined with negatively charged GO to form an integral stable complex (Figure 1a). PPE can bind to A*β* aggregates nonspecifically and can be replaced or self-aggregated, leading to fluorescence recovery or further quenching [28]. Normally, a short fluorescence lifetime and low quantum yield of ThT in water are generally observed as the rapid rotation of C–C bond between two aromatic ring leads to the dissipation of excitation state energy [31]. However, the rotation of ThT is limited by the geometric constraints in the binding site and, thus, exhibits increased quantum yield after binding with fibrotic amyloid protein [32,33]. NR and VBB are also commercial specific probes for amyloid fibrils and show little changes toward monomeric proteins [34,35,36,37].

**Figure 2:**
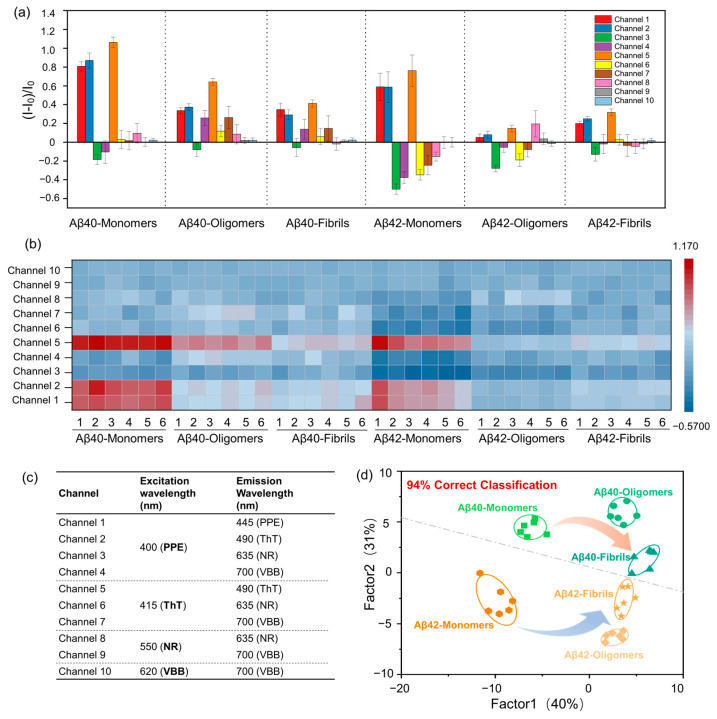
(**a**) Multichannel fluorescence response pattern (I − I_0_)/I_0_ obtained by sensor array against A*β*40/42 aggregates (1 µM) in PBS, error bars indicate the standard deviation (SD) of six replicates. (**b**) Heat map of the fluorescence response of A*β*40/42 aggregates in PBS. Six replicates are shown for each protein. (**c**) The excitation and emission wavelength of each channel. (**d**) Canonical score plot for the first two factors of fluorescence patterns obtained from the sensor array with A*β*40/42 aggregates in PBS. The scores were generated through LDA with 95% confidence ellipses.

In Figure S1 (Table S1), the fluorescence emission peaks of PPE, ThT, NR and VBB were located at 445 nm, 490 nm, 635 nm and 700 nm, respectively. The excitation wavelengths of four dyes were selected for obtaining fluorescent intensities (Figure 2c), leading to 10-channel signal outputs in a single well. To construct the multichannel sensing system, the four dyes mixing solution were mixed with GO. After the addition of GO, the fluorescent emissions were obviously quenched. The ratio of four dyes/GO were selected for the construction of multichannel sensor array when the fluorescence intensity of PPE was quenched to about 70% through the titration experiment (Figure S2).

The fluorescence response of each sensing channel toward A*β*40/A*β*42 (1 µM) with different aggregating states was verified by calculating the relative fluorescence change (I − I_0_)/I_0_ of each signal channel (Figure 2a). Each channel showed various fluorescent intensity changes for A*β*40/A*β*42 with different aggregating states as A*β*40/A*β*42 proteins could selectively bind to one or more dyes by competing with GO. For example, Channel 1, 2, and 5 showed enhanced fluorescence for all A*β*40/A*β*42 species, channel 3 exhibited multifariously quenched fluorescence, while channel 4, 6, and 7 revealed various fluorescent responses for A*β*40/A*β*42 species. In our sensing system, 10-channel signal outputs could be obtained in a single measurement which produced a unique fluorescence response pattern for A*β*40/A*β*42 aggregates, making the rationality of sensor array for the detection of A*β* proteins via the construction of fingerprint. The prominent fluorescent response patterns via cross-reactive responses could also be observed in the heatmap (Figure 2b). Training matrices (10 channels × 6 analytes × 6 replicates) were created, and LDA results were performed using SYSTAT software (Figure S4 and Table S2). In the typical score plot, factor 1 accounted for 40% of the total variance and the sum of factor 1 and factor 2 accounted for 71% of the total variance, which provided the best discrimination among six A*β* aggregate species. A*β*40 was in the upper part of the score chart, and A*β*42 was in the lower part of the score chart (Figure 2d). The cross-validated jackknifed classification matrix showed 94% accuracy (Table S3). To verify the ability of 10-channel sensor array for the prediction of unknown samples, 24 unknown A*β* proteins with different aggregation forms were randomly selected as blind test samples, and all 24 unknown proteins were distinguished with 100% prediction accuracy (Table S4).

**Figure 3:**
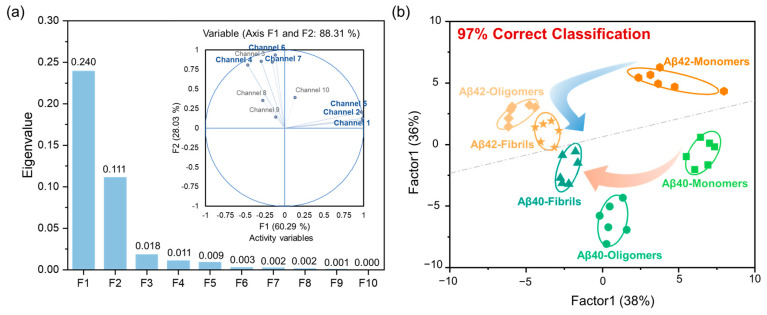
(**a**) Principal component analysis (PCA) contribution plots for channel 1-channel 10. (**b**) Canonical score plot for the first two factors of fluorescence patterns obtained from the optimized 6-channel sensor array with six A*β*40/A*β*42 species in PBS.

The number of sensing channel represents the workload in the detecting process, and thus, the simplification for sensing channels of multichannel sensor array is also necessary through algorithms. The principal component analysis (PCA) approach was employed to remove redundant signal channels from our sensing system. Only the signal channels with high contribution rates were retained within the sensor array, while low-contributing channels were eliminated. According to the output result of PCA, the discriminating contribution of the first two PCs was as high as 88.31% (Figure 3a). Therefore, six channels (channel 1, channel 2, channel 4, channel 5, channel 6 and channel 7) with the largest contribution in the first two PCs were selected to identify and distinguish six A*β*40/A*β*42 species (Figure 3a, Tables S5 and S6). Using the LDA algorithm, it can be seen from the 2D typical score map that A*β*40/A*β*42 species continued to be well divided into six clusters without any misclassification. The clusters of A*β*40 and A*β*42 were distributed on the upper and lower sides of the canonical score plot, respectively (Figure 3b and Figure S5). The cross-validated jackknifed classification matrix showed 97% accuracy and the prediction accuracy for unknown samples was 100% in blind test (Tables S7 and S8). The improved discriminating accuracy indicated that the simplification of sensing channel achieved by PCA screening could remove elements with interfering effects and generate more effective models.

**Figure 4:**
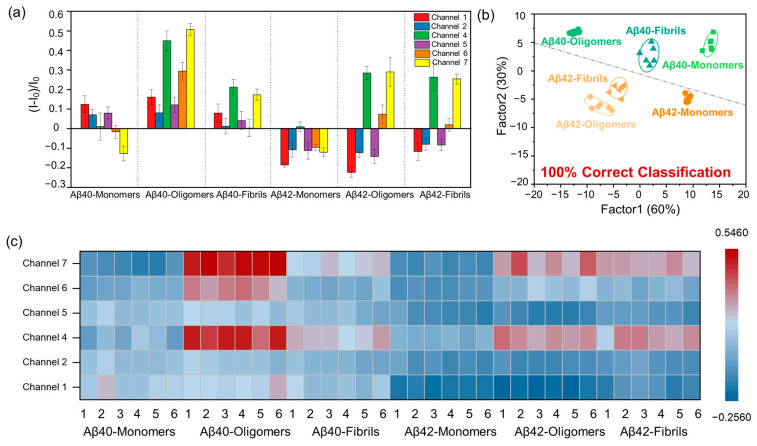
(**a**) Fluorescence response pattern (I−I_0_)/I_0_ obtained by optimized 6-channel sensor array against A*β*40/42 proteins (1 µM) in serum, error bars indicate the standard deviation (SD) of six replicates. (**b**) Canonical score plot for the first two factors of fluorescence patterns obtained from the optimized 6-channel sensor array with A*β*40/42 proteins in serum. The scores were generated through LDA with 95% confidence ellipses. (**c**) Six-channel heat map of the fluorescence response of A*β*40/42 protein in serum. Six replicates are shown for each protein.

Plasma A*β*42/A*β*40 has been reported to directly reflect the accumulation of amyloid plaques in the brains of AD patients, so blood-based test has been used for AD diagnosis [38,39,40,41]. To further verify the practical application capability of our sensing system, the multichannel sensor array was used to discriminate A*β* proteins (1 µM) in serum samples (Figures S3 and S7 and Tables S12–S14). Similarly, each channel produced various fluorescent responses towards A*β*40/A*β*42 species (Figure 4a). Moreover, the distinctive response patterns can be obtained with the heatmap generated from relative fluorescence changes. The training matrix (6 channels × 6 analytes × 6 replicates) was acquired and converted into canonical scores by LDA (Table S9). In the canonical score plot (Figure 4b and Figure S6), various aggregate types of A*β*40/A*β*42 proteins could be clearly visualized, forming six separate clusters. The sum of factor 1 and factor 2 accounted for 90% of the total variance. Meanwhile, the heat map showed the unique response patterns generated through cross-reaction between 6-channel sensor array and A*β*40/A*β*42 (Figure 4c). According to the jackknifed classification matrix, the recognition accuracy for each A*β* aggregate was 100% (Table S10). Twenty-four kinds of *β*-amyloid proteins were randomly selected for blind testing, with 91.7% prediction accuracy, demonstrating the potential of our simplified sensing system in the discrimination of unknown samples (Table S11). These results revealed that the multichannel sensor array had a strong recognition ability for A*β* proteins and the potential for clinical detection of AD.

**Figure 5:**
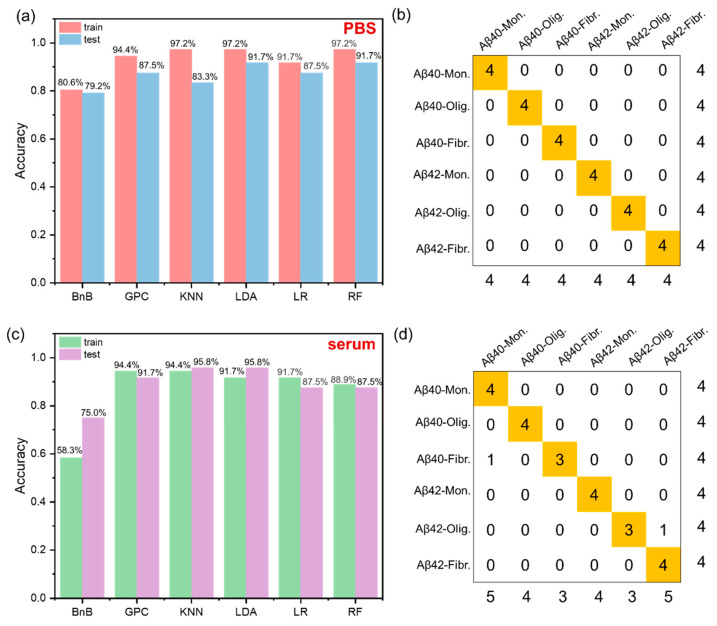
(**a**) Usage of machine learning (ML) methods and classical statistical method LDA for pattern recognition of 1 µM Amyloid-*β* protein by 6 channels in PBS. (**b**) Confusion matrix plot of the output of the classifier from the LDA results by SYSTAT (version 13.0) for unknown sample detection. (**c**) Usage of machine learning (ML) methods and classical statistical method LDA for pattern recognition of 1 µM Amyloid-*β* peptides by 6 channels in serum. (**d**) Confusion matrix plot of the output of the classifier from the KNN results for unknown sample detection.

The accuracy improvement of sensing system is a key factor to achieve practical applications; thus, the approach to improved detecting accuracy is worth exploring. As far as we know, machine learning algorithms have confirmed the strong power in the optimization of sensing results. Therefore, machine learning algorithms including branch and bound (BnB), generalized predictive control (GPC), K-nearest neighbor (KNN), logistic regression (LR) and random forest (RF) were applied to optimize the detecting results of 6-channel sensor array. The dataset we took consisted of 60 examples from different aggregate species of A*β*40/A*β*42 with a ratio of 6:4 (training set:test set). For discriminating A*β* protein in PBS (Figure 5a), LDA and RF algorithms showed the highest training accuracy (97.2%) and test accuracy (91.7%). For the detecting experiments in serum samples, KNN algorithm illustrated the highest training accuracy (94.4%) and test accuracy (95.8%), surpassing the LDA result (Figure 5c).

## 3. Methods and Materials

### 3.1. Reagent

Thioflavin T (E080911) was purchased from energy-chemical. Nile Red (D051404) was purchased from energy-chemical. Victoria Blue B (V820449) and 1,1,1,3,3,3-Hexafluoroisopropanol (HFIP) (H811026) were purchased from Macklin. Beta amyloid 1-40 (107P33) were purchased from Nanjing Peptide Valley Biotechnology Co., Ltd. (Nanjing, China) and beta amyloid 1-42 (A834109) were purchased from Macklin (Shanghai, China). Single-layer GO with a thickness of 0.8–1.2 nm was purchased from XFNANO Materials Tech Co., Ltd. (Nanjing, China). Phosphate-buffered saline (PBS powder, 0.01 M, pH 7.4) was purchased from Beijing Solar Bio-Science & Technology Co., Ltd. (Beijing, China). Human serum was purchased from XINFAN TECHNOLOGY (Shanghai, China). PPE was synthesized according to the reported procedures [19,28,42].

### 3.2. Instrumentation

The fluorescence values were recorded on a SpectraMaxR ID3 Multi-Mode Microplate Reader (Molecular Devices, San Jose, CA, USA), at room temperature. The 96-well plates were produced from Costar (3590, Washington, DC, USA).

### 3.3. Machine Learning Algorithms

Machine learning methods, including branch and bound (BnB), generalized predictive control (GPC), K-nearest neighbor (KNN), logistic regression (LR) and random forest (RF) were built in Python using the scikit-learn package, which is an open-source tool for data analysis and machine learning. (https://github.com/scikit-learn/scikit-learn, accessed on 15 September 2022). The division of the data set calls the ‘train_test_split’ function in scikit-learn, and the test set size is 0.4. (Random state = 4). All test results are cross-validated ten times with ‘cross_val_score’.

### 3.4. Linear Discriminant Analysis

Linear discriminant analysis (LDA) was carried out using classical LDA in SYSTAT (version 13.0, licensed by Systat Software Inc., San Jose, CA, USA). In LDA, all variables were used in the model (complete model) and the tolerance was set as 0.001. The fluorescence response patterns were transformed into canonical patterns. The Mahalanobis distances of each individual pattern to the centroid of each group in a multidimensional space were calculated and the assignment of the case was based on the shortest Mahalanobis distance.

### 3.5. Titration Experiment and Preparation of Sensor

An amount of 1mM PPE stock solution was prepared with deionized water. ThT, NR and VBB stock solutions were prepared by dispersing 5 mM ThT, NR and VBB in DMSO, respectively, and filtered with a 0.22 µM filter. The stock solution of PPE and three dyes (ThT, NR, VBB) was diluted to 8 µM with PBS and then mixed in a ratio of 1:1:1:1. Dilute 1 mg/mL of GO solution with PBS to different concentration gradients. Then, add 100 µL of the mixed solution above to the 96-well plate and add 100 µL of GO solution of different concentrations, respectively. The fluorescence titration curve was read through the microplate reader. (Figure S2) The ratio of four dyes/GO was selected for the construction of multichannel sensor array when the fluorescence intensity of PPE was quenched to about 70%. In order to ensure that the final concentrations of PPE and three dyes were 1 µM, respectively, when reacting with proteins, the stock solutions of PPE and three dyes (ThT, NR, VBB) were diluted to 16 µM with phosphate buffer (10 mM, pH 7.4), respectively. Then, the four 16 µM solutions are mixed in equal volumes to prepare a mixed solution. The final concentration of each dye in the mixed solution is 4 µM. Finally, according to the results of the titration experiment, the mixed solution was mixed with 8 × 10^−4^ mg/mL GO at the corresponding concentration in equal volumes to obtain the sensor.

### 3.6. Pretreatment of Aβ Proteins

According to the literature [43], A*β* monomers powder was completely dissolved in HFIP, placed at room temperature for 6h, and dried in vacuum to remove HFIP. A*β* monomers solution was prepared by dissolving the lyophilized powder of A*β* monomers in PBS (10 mM, pH 7.4) and sodium hydroxide (1 mM) at the desired concentration. The 100 µM A*β*40 monomers solution was prepared by adding 100 µL sodium hydroxide and 130 µL PBS to the lyophilized powder of A*β*40 monomers. Additionally, the 100 µM A*β*42 monomers solution was dissolved by 100 µL sodium hydroxide and 120 µL PBS. To obtain A*β*40 oligomers, 100 µM A*β*40 monomers solution was incubated, at 37 °C, for 12 h by shaking. To obtain A*β*40 fibrils, 100 µM A*β*40 monomers solution was incubated, at 37 °C, for 24 h by shaking. In addition, A*β*42 monomers, A*β*42 oligomers and A*β*42 fibrils were prepared by the same methods.

### 3.7. Aβ40/Aβ42 Aggregates Identification

A*β*40/A*β*42 monomers, A*β*40/A*β*42 oligomers and A*β*40/A*β*42 fibrils were diluted to 2 µM by PBS (10 mM, pH 7.4). Then, 100 µL of sensing solution and 100 µL sample solution of different A*β* aggregation states or phosphate buffer (10 mM sodium phosphate, pH 7.4) for the control experiment were added to each well on a 96-well plate, respectively. The final concentration of A*β* peptides for fluorescence emission measurement is 1 µM. The 96-well plate was incubated, at room temperature, for 60 min. The fluorescence intensity of the array before and after the addition of proteins was recorded by microplate reader and collected by 10-channel fluorescence signals (Figure 2c,d). Finally, the fluorescent data were evaluated by LDA through SYSTAT (version 13.0) (Tables S2–S4). Except for the A*β* protein being diluted with artificial serum to 2 µM, the experimental procedures are the same in experiment of the serum sample (Tables S12–S14).

## 4. Conclusions

In this study, a multichannel fluorescent sensor array composed of four fluorescent dyes and GO through electrostatic and π–π interaction was developed. The single-well five-element complex formed by dyes and GO exhibited pattern responses towards various A*β*40/A*β*42 species through 10-channel signal outputs. Meanwhile, a 6-channel sensor array simplified by the PCA algorithm which showed higher discriminating and predicting performance for PCA screening highlights the contribution of the best sensing channel and removes interference factors such as background noise. Moreover, our sensing system illustrated the detecting ability in the serum. The optimization of the sensing model could be achieved with higher accuracy through machine learning algorithms, demonstrating the power of the sensor array with multichannel signals in clinical detection via machine learning algorithms.

## Data Availability

The data presented in this study are available upon reasonable request from the corresponding author.

## Supplementary Materials

The following supporting information can be downloaded at: https://www.mdpi.com/article/10.3390/ijms232314562/s1.
