# Supplementary material for: A Multichannel Fluorescent Tongue for Amyloid-β Aggregates Detection
Source: Int J Mol Sci. 2022 Nov 23;23(23):14562. doi: 10.3390/ijms232314562 (PMC9739152; doi:10.3390/ijms232314562)
Supplement: Supplementary file 1 [file ijms-23-14562-s001.zip › ijms-2002125-supplementary.pdf]

Supporting Information

A Multichannel Fluorescent Tongue for Amyloid-*β* Aggregates Detection

Table of Contents

1. General Information and Fluorescence spectra S2
2. Statistical Analysis S3

1. General Information and Fluorescence spectra

Supplementary Table S1 Excitation and emission wavelengths of 4 dyes.

Dyes	Excitation wavelength	Emission wavelength
PPE	400 nm	445 nm
ThT	415 nm	490 nm
NR	550 nm	635 nm
VBB	620 nm	700 nm

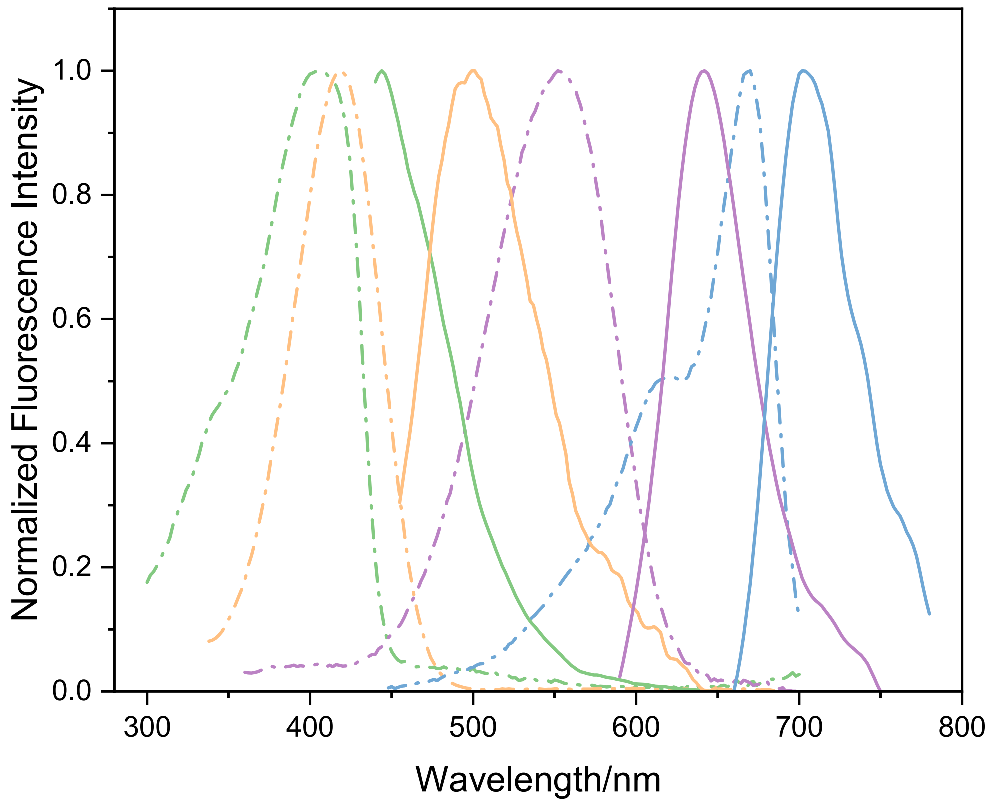

Supplementary Figure S1 Normalized excitation (dotted line) and emission spectra (solid line) of PPE (green), ThT (yellow) NR (purple) and VBB (blue).

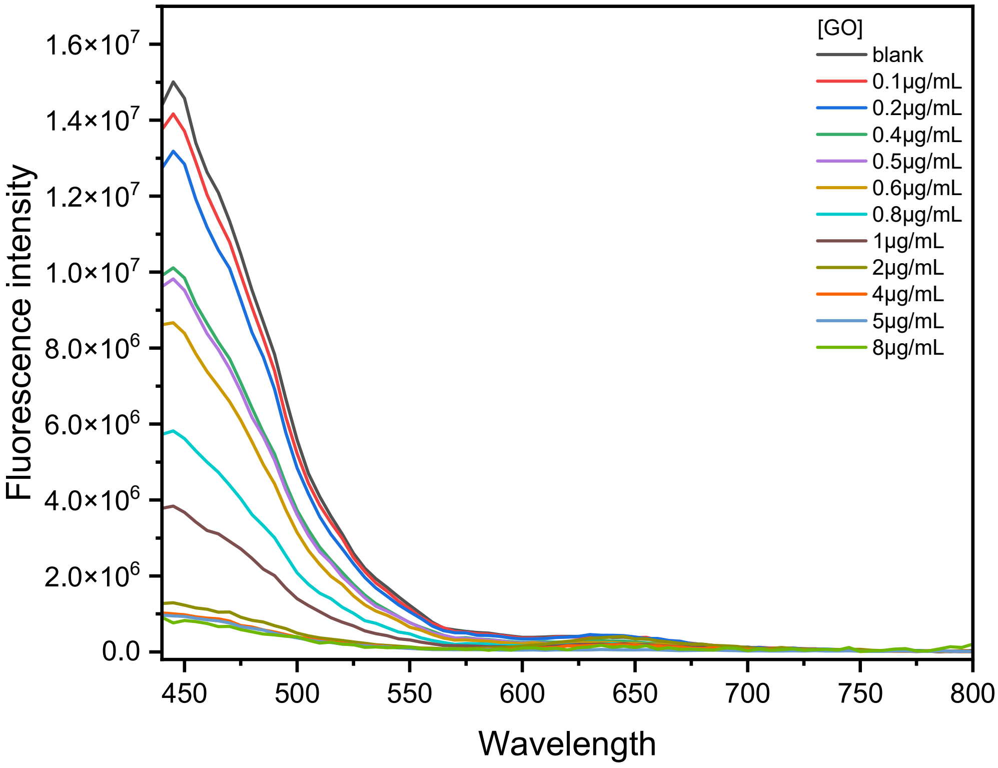

Supplementary Figure S2 Fluorescence quenching curves of PPE, ThT, NR and VBB mixed solution titrated with different concentrations of graphene oxide (GO) in buffer (λex =400 nm). The concentration of four dyes after mixing is 4 μM.

2. Statistical Analysis

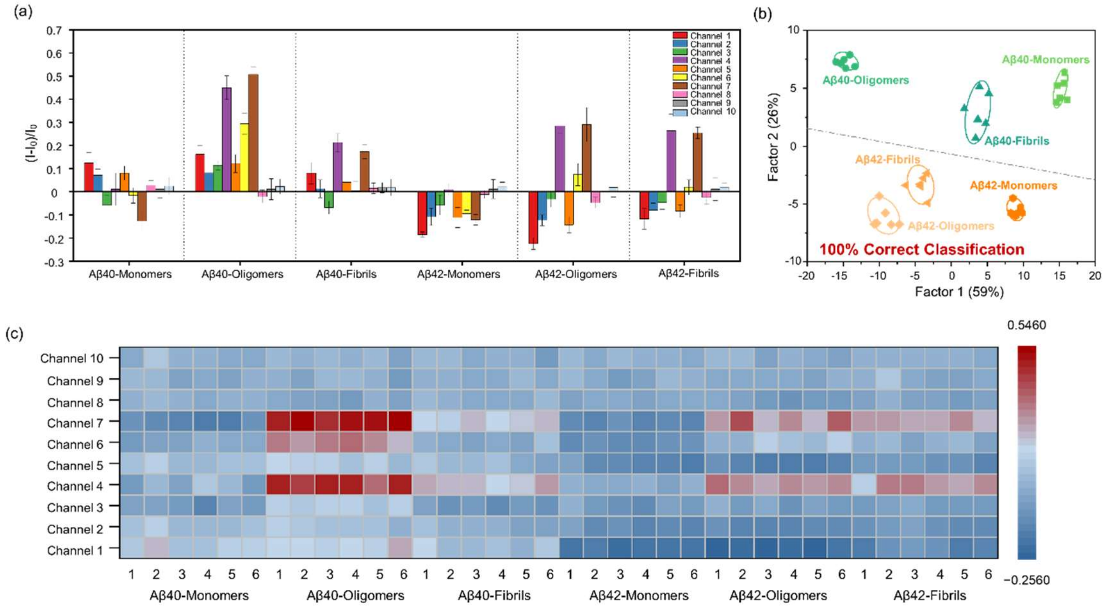

Supplementary Figure S3 (a) 10-channel fluorescence response of sensor array against A*β*40/42 protein in serum, error bars indicate the standard deviation (SD) of six replicate

protein. (b) Canonical score plot for the first two factors of fluorescence patterns obtained from the 10-channel sensor array with A*β*40/42 protein in serum. The scores were generated through LDA with 95% confidence ellipses. (c) Heat map of the fluorescence response by 10 channels of A*β*40/42 protein in serum. Six replicates are shown for each protein.

Supplementary Table S2 Training matrix of fluorescence response pattern from an array of channel 1-channel 10 against 1μM Amyloid-β peptides in buffer. LDA was carried out and resulted in 3 factors of the canonical scores.

Analyte	Fluorescence response pattern										Result LDA			
Aβ peptides	channel 1	channel 2	channel 3	channel 4	channel 5	channel 6	channel 7	channel 8	channel 9	channel 10	Factor 1	Factor 2	Factor 3	Group
Aβ40-monomers	0.81	0.82	-0.19	-0.06	1.04	0.15	0.00	0.05	-0.01	0.00	-5.77	5.34	2.51	3
Aβ40-monomers	0.88	1.00	-0.14	-0.19	1.17	0.02	0.07	0.11	-0.02	0.02	-6.92	4.63	2.27	3
Aβ40-monomers	0.77	0.85	-0.13	-0.11	1.02	0.02	0.12	0.17	0.07	0.01	-4.51	3.77	1.04	3
Aβ40-monomers	0.74	0.81	-0.17	0.11	1.03	0.12	-0.14	0.23	0.05	0.05	-5.88	4.95	0.96	3
Aβ40-monomers	0.80	0.81	-0.27	-0.13	1.04	-0.11	-0.05	0.09	-0.05	0.00	-7.31	4.02	0.66	3
Aβ40-monomers	0.85	0.93	-0.22	-0.23	1.08	-0.03	0.09	-0.08	-0.02	0.04	-6.60	3.53	2.45	3
Aβ40-Oligomers	0.29	0.35	0.01	0.24	0.62	0.10	0.13	0.23	0.02	-0.01	3.66	4.70	-2.93	2
Aβ40-Oligomers	0.33	0.41	-0.19	0.33	0.67	0.10	0.27	0.08	0.03	0.05	2.26	5.55	-3.91	2
Aβ40-Oligomers	0.32	0.34	-0.10	0.38	0.63	0.21	0.33	0.17	0.03	0.04	3.99	7.07	-3.49	2
Aβ40-Oligomers	0.37	0.37	-0.12	0.19	0.67	0.16	0.38	0.05	0.06	0.00	2.59	6.59	-3.68	2
Aβ40-Oligomers	0.33	0.34	-0.05	0.22	0.59	0.03	0.36	0.04	0.01	0.03	5.05	5.62	-2.85	2
Aβ40-Oligomers	0.39	0.43	-0.02	0.19	0.67	0.12	0.11	-0.06	-0.03	0.03	3.06	5.40	-0.65	2
Aβ40-Fibrils	0.30	0.21	-0.20	0.10	0.35	-0.11	0.11	-0.07	0.00	0.02	4.52	-0.08	0.26	1
Aβ40-Fibrils	0.31	0.29	-0.01	0.10	0.40	0.11	0.28	-0.02	0.00	0.04	6.85	1.99	1.60	1
Aβ40-Fibrils	0.34	0.28	-0.03	0.35	0.44	0.07	0.01	0.09	0.04	0.02	6.58	2.06	1.35	1
Aβ40-Fibrils	0.37	0.36	0.07	0.10	0.46	0.06	0.00	0.01	0.02	-0.02	6.43	0.35	3.41	1
Aβ40-Fibrils	0.28	0.28	-0.03	0.07	0.40	0.13	0.33	-0.04	0.01	0.03	6.40	2.18	0.77	1
Aβ40-Fibrils	0.47	0.33	-0.14	0.12	0.43	0.09	0.14	-0.10	0.02	0.05	4.75	1.55	4.01	1
Aβ42-monomers	0.84	0.86	-0.48	-0.43	1.07	-0.39	-0.12	-0.21	0.01	-0.03	-11.64	-0.03	-0.36	6
Aβ42-monomers	0.61	0.64	-0.42	-0.31	0.82	-0.34	-0.28	-0.14	0.05	0.02	-9.44	-1.86	-1.21	6
Aβ42-monomers	0.59	0.54	-0.54	-0.31	0.65	-0.34	-0.21	-0.15	0.05	0.06	-8.54	-3.66	0.28	6

Aβ42-monomers	0.58	0.63	-0.47	-0.45	0.75	-0.25	-0.29	-0.07	0.02	0.04	-10.73	-3.74	0.42	6
Aβ42-monomers	0.51	0.47	-0.52	-0.43	0.67	-0.35	-0.17	-0.17	-0.12	-0.07	-8.16	-2.75	-0.69	6
Aβ42-monomers	0.40	0.38	-0.57	-0.33	0.61	-0.42	-0.41	-0.18	-0.03	-0.02	-9.59	-4.02	-2.77	6
Aβ42-Oligomers	0.02	0.07	-0.24	-0.05	0.12	-0.08	-0.05	0.23	0.06	0.00	3.23	-6.20	-1.16	5
Aβ42-Oligomers	0.03	0.03	-0.29	-0.04	0.11	-0.28	-0.07	-0.06	0.00	0.03	3.63	-5.68	-1.72	5
Aβ42-Oligomers	0.03	0.09	-0.31	-0.12	0.13	-0.21	-0.04	0.35	0.02	-0.01	1.70	-6.85	-2.07	5
Aβ42-Oligomers	0.11	0.13	-0.23	-0.11	0.21	-0.18	-0.11	0.19	0.12	-0.02	1.81	-6.16	-1.65	5
Aβ42-Oligomers	0.05	0.05	-0.27	-0.04	0.15	-0.24	-0.22	0.18	-0.05	-0.05	2.46	-5.92	-1.23	5
Aβ42-Oligomers	0.07	0.11	-0.32	0.03	0.16	-0.15	0.01	0.27	0.06	-0.04	3.56	-6.52	-1.45	5
Aβ42-Fibrils	0.19	0.27	0.00	-0.02	0.37	0.03	-0.11	-0.02	-0.02	0.05	3.54	-1.38	1.04	4
Aβ42-Fibrils	0.22	0.23	-0.17	0.10	0.30	0.03	-0.03	-0.11	0.00	0.02	4.92	-2.44	2.08	4
Aβ42-Fibrils	0.16	0.23	-0.16	-0.04	0.27	0.02	-0.02	-0.01	-0.10	0.03	3.67	-2.96	1.93	4
Aβ42-Fibrils	0.21	0.23	-0.19	0.05	0.31	-0.06	-0.21	-0.03	-0.03	0.00	2.94	-3.46	1.76	4
Aβ42-Fibrils	0.23	0.28	-0.09	0.00	0.36	0.12	0.05	0.05	-0.03	0.01	4.11	-1.33	1.85	4
Aβ42-Fibrils	0.18	0.25	-0.16	-0.20	0.29	0.03	0.12	-0.15	0.06	-0.02	3.40	-4.24	1.17	4

Supplementary Table S3 LDA jackknifed classification matrix table obtained from the array of channel 1-channel 10 against 1μM Amyloid-β peptides in buffer. The jackknifed classification matrix with cross-validation reveals a 94% accuracy.

	Aβ40-Fibrils	Aβ40-Oligomers	Aβ40-monomers	Aβ42-Fibrils	Aβ42-Oligomers	Aβ42-monomers	%Correct
Aβ40-Fibrils	6	0	0	0	0	0	100
Aβ40-Oligomers	0	6	0	0	0	0	100
Aβ40-monomers	0	0	6	0	0	0	100
Aβ42-Fibrils	0	0	0	6	0	0	100
Aβ42-Oligomers	0	0	0	1	5	0	83
Aβ42-monomers	0	0	1	0	0	5	83
Total	6	6	7	7	5	5	94

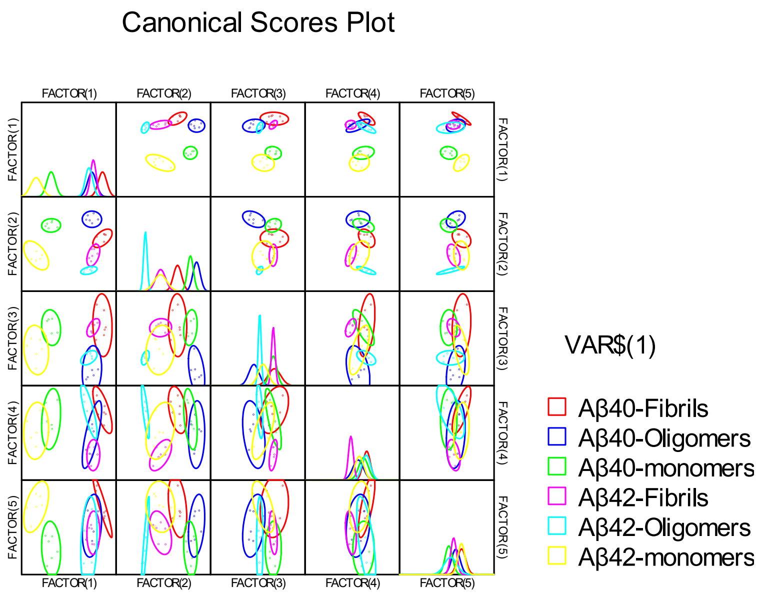

Supplementary Figure S4 Correlations of canonical fluorescence response patterns from an array of 10 channels against 1μM Amyloid-β peptides in PBS. The 95% confidence ellipses for the individual analytes are shown.

Supplementary Table S4 Detection and identification of unknown samples in water using LDA from the array of channel 1-channel 10. According to the verification, 24 among 24 unknown samples were correctly identified, representing an accuracy of 100%.

Analyte	Fluorescence response pattern										Results LDA					
Unknown samples	channel 1	channel 2	channel 3	channel 4	channel 5	channel 6	channel 7	channel 8	channel 9	channel 10	Factor 1	Factor 2	Factor 3	Group	Identification	Verification
1	0.89	0.95	-0.14	-0.09	1.14	-0.03	0.22	0.00	0.01	0.04	-4.68	6.04	1.43	3	Aβ40-monomers	Aβ40-monomers

2	0.89	0.92	-0.16	-0.11	1.19	0.12	0.15	0.08	-0.03	0.01	-6.98	8.06	1.00	3	Aβ40-monomers	Aβ40-monomers
3	0.80	0.84	-0.14	-0.16	1.06	0.02	-0.02	-0.04	-0.05	0.02	-5.99	5.25	2.10	3	Aβ40-monomers	Aβ40-monomers
4	0.85	0.89	-0.09	-0.10	1.05	0.03	0.20	0.13	0.01	0.01	-3.39	5.23	2.46	3	Aβ40-monomers	Aβ40-monomers
5	0.39	0.41	-0.06	0.07	0.63	0.23	0.34	-0.03	-0.01	0.02	3.01	5.49	-0.44	2	Aβ40-Oligomers	Aβ40-Oligomers
6	0.36	0.36	-0.12	0.01	0.66	0.10	0.27	-0.02	0.01	0.03	0.15	5.79	-3.47	2	Aβ40-Oligomers	Aβ40-Oligomers
7	0.37	0.39	0.06	0.09	0.66	0.15	0.35	-0.01	0.03	0.04	3.87	6.87	-2.23	2	Aβ40-Oligomers	Aβ40-Oligomers
8	0.34	0.35	-0.11	0.10	0.57	0.16	0.24	0.06	-0.02	-0.01	2.78	4.10	-0.99	2	Aβ40-Oligomers	Aβ40-Oligomers
9	0.38	0.28	0.00	0.02	0.34	0.16	0.24	0.03	0.06	0.02	6.74	0.58	3.88	1	Aβ40-Fibrils	Aβ40-Fibrils
10	0.21	0.15	-0.11	0.03	0.25	-0.09	0.19	0.02	0.00	0.06	5.79	-0.47	0.00	1	Aβ40-Fibrils	Aβ40-Fibrils
11	0.24	0.16	-0.15	-0.01	0.28	-0.02	0.03	0.04	0.04	0.02	3.56	-1.37	0.69	1	Aβ40-Fibrils	Aβ40-Fibrils
12	0.30	0.18	-0.10	0.04	0.29	0.05	0.24	0.06	0.03	0.03	5.72	0.48	1.36	1	Aβ40-Fibrils	Aβ40-Fibrils
13	0.55	0.50	-0.46	-0.22	0.67	-0.33	-0.23	-0.14	0.02	0.03	-6.96	-2.22	-0.39	6	Aβ42-monomers	Aβ42-monomers
14	0.48	0.43	-0.46	-0.31	0.62	-0.37	-0.31	-0.19	-0.05	-0.03	-7.16	-3.04	-0.66	6	Aβ42-monomers	Aβ42-monomers

15	0.53	0.58	-0.49	-0.29	0.77	-0.31	-0.15	-0.15	0.02	0.01	-8.63	-1.88	-2.33	6	Aβ42-monomers	Aβ42-monomers
16	0.58	0.56	-0.49	-0.30	0.76	-0.29	-0.09	-0.16	-0.04	0.00	-8.02	-0.67	-1.19	6	Aβ42-monomers	Aβ42-monomers
17	0.12	0.13	-0.24	-0.14	0.20	-0.18	0.01	0.04	-0.02	-0.03	3.19	-4.95	-0.30	5	Aβ42-Oligomers	Aβ42-Oligomers
18	0.08	0.12	-0.26	-0.08	0.17	-0.18	0.21	0.10	-0.02	0.00	4.88	-4.53	-1.31	5	Aβ42-Oligomers	Aβ42-Oligomers
19	0.01	0.02	-0.26	-0.22	0.12	-0.14	0.19	0.17	0.02	-0.02	2.99	-4.81	-2.95	5	Aβ42-Oligomers	Aβ42-Oligomers
20	0.04	0.06	-0.19	-0.15	0.10	-0.04	0.04	0.16	0.02	-0.04	4.42	-5.98	0.12	5	Aβ42-Oligomers	Aβ42-Oligomers
21	0.17	0.22	-0.06	-0.08	0.27	0.03	-0.03	-0.10	0.03	0.02	4.63	-3.20	1.90	4	Aβ42-Fibrils	Aβ42-Fibrils
22	0.16	0.23	-0.17	0.03	0.27	-0.02	0.10	-0.03	0.00	-0.02	5.59	-3.61	0.98	4	Aβ42-Fibrils	Aβ42-Fibrils
23	0.20	0.25	-0.02	-0.15	0.32	0.03	-0.01	-0.06	0.00	-0.01	4.16	-2.61	1.87	4	Aβ42-Fibrils	Aβ42-Fibrils
24	0.13	0.21	-0.10	0.00	0.31	-0.04	0.04	-0.04	-0.03	0.01	4.85	-2.11	-0.09	4	Aβ42-Fibrils	Aβ42-Fibrils

Supplementary Table S5 Principal component analysis of test results for 1μM Amyloid-β peptides in buffer.

	F1	F2	F3	F4	F5
channel 1	26.689	0.596	0.390	0.544	0.216
channel 2	28.845	0.970	0.582	0.215	3.879
channel 3	0.955	17.284	0.293	14.679	21.155

channel 4	4.242	27.250	2.430	5.838	53.056
channel 5	38.061	3.663	1.068	0.375	6.103
channel 6	0.188	24.583	0.306	7.386	10.875
channel 7	0.371	23.232	15.148	59.186	0.310
channel 8	0.631	2.253	78.862	10.032	4.315
channel 9	0.012	0.039	0.772	1.433	0.001
channel 10	0.007	0.131	0.149	0.312	0.090

Supplementary Table S6 Training matrix of fluorescence response pattern from an array of channels screened by PCA against 1μM Amyloid-β peptides in buffer. LDA was carried out and resulted in 3 factors of the canonical scores.

Analyte	Fluorescence response pattern						Result LDA			
Aβ peptides	channel 1	channel 2	channel 4	channel 5	channel 6	channel 7	Factor 1	Factor 2	Factor 3	Group
Aβ40-monomers	0.81	0.82	-0.06	1.04	0.15	0.00	6.92	-1.66	2.41	3
Aβ40-monomers	0.88	1.00	-0.19	1.17	0.02	0.07	7.35	-0.19	1.62	3
Aβ40-monomers	0.77	0.85	-0.11	1.02	0.02	0.12	5.49	-0.99	1.36	3
Aβ40-monomers	0.74	0.81	0.11	1.03	0.12	-0.14	6.05	-2.04	1.69	3
Aβ40-monomers	0.80	0.81	-0.13	1.04	-0.11	-0.05	7.01	0.10	0.11	3
Aβ40-monomers	0.85	0.93	-0.23	1.08	-0.03	0.09	6.44	0.57	1.73	3
Aβ40-Oligomers	0.29	0.35	0.24	0.62	0.10	0.13	0.48	-5.04	-2.56	2
Aβ40-Oligomers	0.33	0.41	0.33	0.67	0.10	0.27	0.39	-6.72	-2.63	2
Aβ40-Oligomers	0.32	0.34	0.38	0.63	0.21	0.33	0.27	-8.08	-1.90	2
Aβ40-Oligomers	0.37	0.37	0.19	0.67	0.16	0.38	1.59	-6.93	-2.44	2
Aβ40-Oligomers	0.33	0.34	0.22	0.59	0.03	0.36	-0.18	-5.79	-2.40	2
Aβ40-Oligomers	0.39	0.43	0.19	0.67	0.12	0.11	1.34	-4.33	-1.10	2
Aβ40-Fibrils	0.30	0.21	0.10	0.35	-0.11	0.11	-2.62	-0.87	0.29	1
Aβ40-Fibrils	0.31	0.29	0.10	0.40	0.11	0.28	-2.76	-2.72	1.63	1

Aβ40-Fibrils	0.34	0.28	0.35	0.44	0.07	0.01	-2.21	-3.20	1.68	1
Aβ40-Fibrils	0.37	0.36	0.10	0.46	0.06	0.00	-1.77	-0.58	2.51	1
Aβ40-Fibrils	0.28	0.28	0.07	0.40	0.13	0.33	-2.64	-3.15	0.95	1
Aβ40-Fibrils	0.47	0.33	0.12	0.43	0.09	0.14	-1.62	-1.48	4.33	1
Aβ42-monomers	0.84	0.86	-0.43	1.07	-0.39	-0.12	7.96	4.33	-1.45	6
Aβ42-monomers	0.61	0.64	-0.31	0.82	-0.34	-0.28	4.74	4.68	-1.60	6
Aβ42-monomers	0.59	0.54	-0.31	0.65	-0.34	-0.21	2.36	5.43	0.64	6
Aβ42-monomers	0.58	0.63	-0.45	0.75	-0.25	-0.29	3.76	6.27	0.22	6
Aβ42-monomers	0.51	0.47	-0.43	0.67	-0.35	-0.17	3.65	4.94	-2.27	6
Aβ42-monomers	0.40	0.38	-0.33	0.61	-0.42	-0.41	3.14	5.66	-3.52	6
Aβ42-Oligomers	0.02	0.07	-0.05	0.12	-0.08	-0.05	-6.14	2.12	-0.25	5
Aβ42-Oligomers	0.03	0.03	-0.04	0.11	-0.28	-0.07	-6.03	2.82	-1.79	5
Aβ42-Oligomers	0.03	0.09	-0.12	0.13	-0.21	-0.04	-5.92	3.07	-1.27	5
Aβ42-Oligomers	0.11	0.13	-0.11	0.21	-0.18	-0.11	-4.40	3.03	-0.82	5
Aβ42-Oligomers	0.05	0.05	-0.04	0.15	-0.24	-0.22	-4.79	3.21	-1.75	5
Aβ42-Oligomers	0.07	0.11	0.03	0.16	-0.15	0.01	-6.16	1.46	-0.44	5
Aβ42-Fibrils	0.19	0.27	-0.02	0.37	0.03	-0.11	-2.85	1.13	0.33	4
Aβ42-Fibrils	0.22	0.23	0.10	0.30	0.03	-0.03	-4.05	0.21	1.82	4
Aβ42-Fibrils	0.16	0.23	-0.04	0.27	0.02	-0.02	-4.41	1.40	1.17	4
Aβ42-Fibrils	0.21	0.23	0.05	0.31	-0.06	-0.21	-3.35	1.91	1.08	4
Aβ42-Fibrils	0.23	0.28	0.00	0.36	0.12	0.05	-3.15	-0.24	1.59	4
Aβ42-Fibrils	0.18	0.25	-0.20	0.29	0.03	0.12	-3.88	1.66	1.03	4

Supplementary Table S7 LDA jackknifed classification matrix table obtained from the array of channels screened by PCA against 1μM Amyloid-β peptides in buffer. The jackknifed classification matrix with cross-validation reveals a 97% accuracy.

	Aβ40-Fibrils	Aβ40-Oligomers	Aβ40-monomers	Aβ42-Fibrils	Aβ42-Oligomers	Aβ42-monomers	%Correct

Aβ40-Fibrils	6	0	0	0	0	0	100
Aβ40-Oligomers	0	6	0	0	0	0	100
Aβ40-monomers	0	0	6	0	0	0	100
Aβ42-Fibrils	0	0	0	6	0	0	100
Aβ42-Oligomers	0	0	0	0	6	0	100
Aβ42-monomers	0	0	1	0	0	5	83
Total	6	6	7	6	6	5	97

Canonical Scores Plot

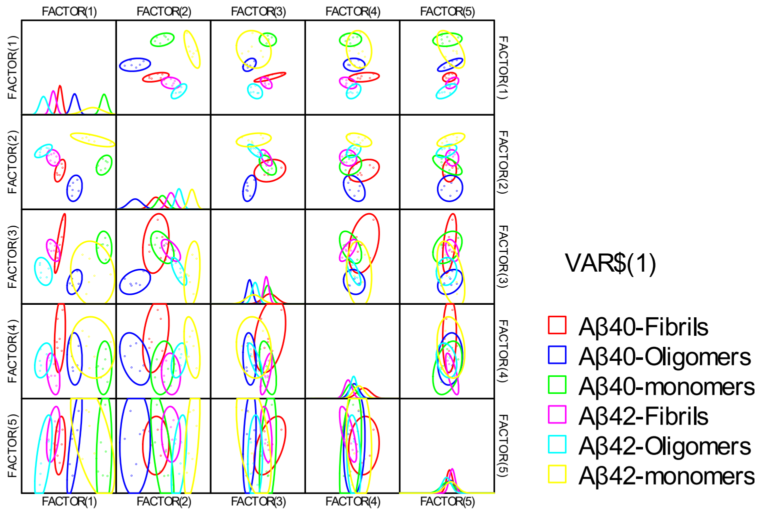

Supplementary Figure S5 Correlations of canonical fluorescence response patterns from an array of 6 channels screened by PCA against 1μM Amyloid-β peptides in PBS. The 95% confidence ellipses for the individual analytes are shown.

Supplementary Table S8 Detection and identification of unknown samples in buffer using LDA from the array of channels screened by PCA against 1μM Amyloid-β peptides in buffer. According to the verification, 24 among 24 unknown samples were correctly identified, representing an accuracy of 100%.

Analyte	Fluorescence response pattern						Results LDA					
Unknown samples	channel 1	channel 2	channel 4	channel 5	channel 6	channel 7	Factor 1	Factor 2	Factor 3	Group	Identification	Verification
1	0.89	0.95	-0.09	1.14	-0.03	0.22	6.83	-1.86	1.11	3	Aβ40-monomers	Aβ40-monomers
2	0.89	0.92	-0.11	1.19	0.12	0.15	8.94	-2.94	0.84	3	Aβ40-monomers	Aβ40-monomers
3	0.80	0.84	-0.16	1.06	0.02	-0.02	7.19	-0.31	1.09	3	Aβ40-monomers	Aβ40-monomers
4	0.85	0.89	-0.10	1.05	0.03	0.20	5.94	-1.57	2.17	3	Aβ40-monomers	Aβ40-monomers
5	0.39	0.41	0.07	0.63	0.23	0.34	0.94	-5.08	-0.12	2	Aβ40-Oligomers	Aβ40-Oligomers
6	0.36	0.36	0.01	0.66	0.10	0.27	2.30	-4.56	-2.87	2	Aβ40-Oligomers	Aβ40-Oligomers
7	0.37	0.39	0.09	0.66	0.15	0.35	1.40	-5.54	-1.95	2	Aβ40-Oligomers	Aβ40-Oligomers
8	0.34	0.35	0.10	0.57	0.16	0.24	0.31	-4.31	-0.68	2	Aβ40-Oligomers	Aβ40-Oligomers
9	0.38	0.28	0.02	0.34	0.16	0.24	-2.94	-1.24	4.30	1	Aβ40-Fibrils	Aβ40-Fibrils
10	0.21	0.15	0.03	0.25	-0.09	0.19	-4.15	-0.44	0.20	1	Aβ40-Fibrils	Aβ40-Fibrils
11	0.24	0.16	-0.01	0.28	-0.02	0.03	-2.99	0.38	1.13	1	Aβ40-Fibrils	Aβ40-Fibrils
12	0.30	0.18	0.04	0.29	0.05	0.24	-3.24	-1.45	2.08	1	Aβ40-Fibrils	Aβ40-Fibrils
13	0.55	0.50	-0.22	0.67	-0.33	-0.23	2.70	4.15	-0.57	6	Aβ42-monomers	Aβ42-monomers
14	0.48	0.43	-0.31	0.62	-0.37	-0.31	2.93	5.17	-1.88	6	Aβ42-monomers	Aβ42-monomers
15	0.53	0.58	-0.29	0.77	-0.31	-0.15	3.70	3.45	-2.53	6	Aβ42-monomers	Aβ42-monomers
16	0.58	0.56	-0.30	0.76	-0.29	-0.09	4.11	3.04	-1.73	6	Aβ42-monomers	Aβ42-monomers
17	0.12	0.13	-0.14	0.20	-0.18	0.01	-4.59	2.60	-0.75	5	Aβ42-Oligomers	Aβ42-Oligomers
18	0.08	0.12	-0.08	0.17	-0.18	0.21	-6.12	1.06	-1.12	5	Aβ42-Oligomers	Aβ42-Oligomers
19	0.01	0.02	-0.22	0.12	-0.14	0.19	-5.42	1.52	-2.25	5	Aβ42-Oligomers	Aβ42-Oligomers
20	0.04	0.06	-0.15	0.10	-0.04	0.04	-6.00	2.33	0.29	5	Aβ42-Oligomers	Aβ42-Oligomers
21	0.17	0.22	-0.08	0.27	0.03	-0.03	-4.08	1.71	1.41	4	Aβ42-Fibrils	Aβ42-Fibrils

22	0.16	0.23	0.03	0.27	-0.02	0.10	-5.05	0.29	0.71	4	Aβ42-Fibrils	Aβ42-Fibrils
23	0.20	0.25	-0.15	0.32	0.03	-0.01	-3.10	1.76	0.93	4	Aβ42-Fibrils	Aβ42-Fibrils
24	0.13	0.21	0.00	0.31	-0.04	0.04	-3.97	0.25	-0.74	4	Aβ42-Fibrils	Aβ42-Fibrils

Supplementary Table S9 Training matrix of fluorescence response pattern from an array of channels screened by PCA against 1μM Amyloid-β peptides in serum. LDA was carried out and resulted in 3 factors of the canonical scores.

Analyte	Fluorescence response pattern						Result LDA			
Aβ peptides	channel 1	channel 2	channel 4	channel 5	channel 6	channel 7	Factor 1	Factor 2	Factor 3	Group
Aβ40-monomers	0.11	0.07	-0.05	0.07	-0.07	-0.08	13.44	4.07	-0.15	3
Aβ40-monomers	0.21	0.12	0.06	0.14	-0.03	-0.12	13.83	6.81	0.00	3
Aβ40-monomers	0.09	0.04	-0.03	0.05	-0.03	-0.13	13.57	3.76	-0.89	3
Aβ40-monomers	0.09	0.06	0.11	0.06	0.03	-0.17	11.78	3.54	-1.83	3
Aβ40-monomers	0.13	0.06	0.04	0.09	0.00	-0.17	13.30	5.10	-0.63	3
Aβ40-monomers	0.13	0.08	-0.07	0.08	0.00	-0.09	13.43	5.24	-1.54	3
Aβ40-Oligomers	0.16	0.14	0.47	0.17	0.32	0.49	-12.56	6.92	-1.95	2
Aβ40-Oligomers	0.15	0.10	0.43	0.14	0.28	0.54	-12.81	6.45	-0.64	2
Aβ40-Oligomers	0.14	0.07	0.47	0.13	0.31	0.46	-12.33	6.58	-0.85	2
Aβ40-Oligomers	0.17	0.07	0.49	0.09	0.35	0.51	-13.38	6.66	-2.13	2
Aβ40-Oligomers	0.12	0.08	0.35	0.14	0.29	0.50	-11.53	6.75	-1.02	2
Aβ40-Oligomers	0.23	0.02	0.48	0.06	0.22	0.55	-11.68	7.32	2.20	2
Aβ40-Fibrils	0.15	0.07	0.24	0.11	0.04	0.17	2.15	5.13	1.51	1
Aβ40-Fibrils	0.04	0.02	0.21	0.05	-0.01	0.12	2.65	1.77	1.60	1
Aβ40-Fibrils	0.06	0.02	0.22	0.04	-0.04	0.20	1.17	1.81	2.75	1
Aβ40-Fibrils	0.09	0.02	0.16	0.08	0.01	0.16	2.52	3.90	1.98	1
Aβ40-Fibrils	0.03	-0.03	0.18	-0.01	-0.04	0.18	1.81	0.92	2.26	1

Aβ40-Fibrils	0.11	-0.03	0.27	-0.02	0.07	0.20	-0.08	3.12	1.23	1
Aβ42-monomers	-0.18	-0.04	0.02	-0.03	-0.10	-0.11	8.54	-3.91	-0.18	6
Aβ42-monomers	-0.17	-0.12	0.00	-0.12	-0.09	-0.13	9.55	-4.47	-0.32	6
Aβ42-monomers	-0.20	-0.10	-0.01	-0.11	-0.11	-0.09	8.69	-5.47	-0.48	6
Aβ42-monomers	-0.19	-0.13	0.04	-0.13	-0.11	-0.14	9.17	-5.35	0.17	6
Aβ42-monomers	-0.18	-0.12	-0.02	-0.13	-0.08	-0.11	9.26	-4.67	-0.82	6
Aβ42-monomers	-0.18	-0.14	0.03	-0.15	-0.07	-0.15	9.03	-4.98	-0.67	6
Aβ42-Oligomers	-0.24	-0.08	0.34	-0.08	0.03	0.27	-7.91	-6.34	0.50	5
Aβ42-Oligomers	-0.22	-0.13	0.28	-0.15	0.01	0.39	-9.11	-6.60	1.14	5
Aβ42-Oligomers	-0.25	-0.14	0.24	-0.17	0.13	0.20	-5.88	-6.45	-2.80	5
Aβ42-Oligomers	-0.21	-0.15	0.28	-0.17	0.08	0.30	-7.52	-5.91	-0.71	5
Aβ42-Oligomers	-0.24	-0.12	0.27	-0.16	0.06	0.22	-5.34	-6.96	-1.46	5
Aβ42-Oligomers	-0.18	-0.11	0.29	-0.13	0.13	0.36	-9.38	-4.54	-1.20	5
Aβ42-Fibrils	-0.17	-0.08	0.14	-0.06	0.03	0.27	-3.89	-3.39	-0.05	4
Aβ42-Fibrils	-0.09	-0.05	0.32	-0.07	0.06	0.26	-4.76	-2.64	0.13	4
Aβ42-Fibrils	-0.07	-0.04	0.33	-0.06	-0.02	0.25	-3.39	-2.84	1.91	4
Aβ42-Fibrils	-0.08	-0.09	0.26	-0.10	0.02	0.24	-3.00	-2.54	0.86	4
Aβ42-Fibrils	-0.15	-0.10	0.24	-0.09	0.04	0.29	-5.81	-3.79	0.57	4
Aβ42-Fibrils	-0.15	-0.12	0.29	-0.13	-0.02	0.22	-3.54	-5.01	1.52	4

Supplementary Table S10 LDA jackknifed classification matrix table obtained from the array of channels screened by PCA against 1μM Amyloid-β peptides in serum. The jackknifed classification matrix with cross-validation reveals a 100% accuracy.

	Aβ40-Fibrils	Aβ40-Oligomers	Aβ40-monomers	Aβ42-Fibrils	Aβ42-Oligomers	Aβ42-monomers	%Correct
Aβ40-Fibrils	6	0	0	0	0	0	100
Aβ40-Oligomers	0	6	0	0	0	0	100
Aβ40-monomers	0	0	6	0	0	0	100

Aβ42-Fibrils	0	0	0	6	0	0	100
Aβ42-Oligomers	0	0	0	0	6	0	100
Aβ42-monomers	0	0	0	0	0	6	100
Total	6	6	6	6	6	6	100

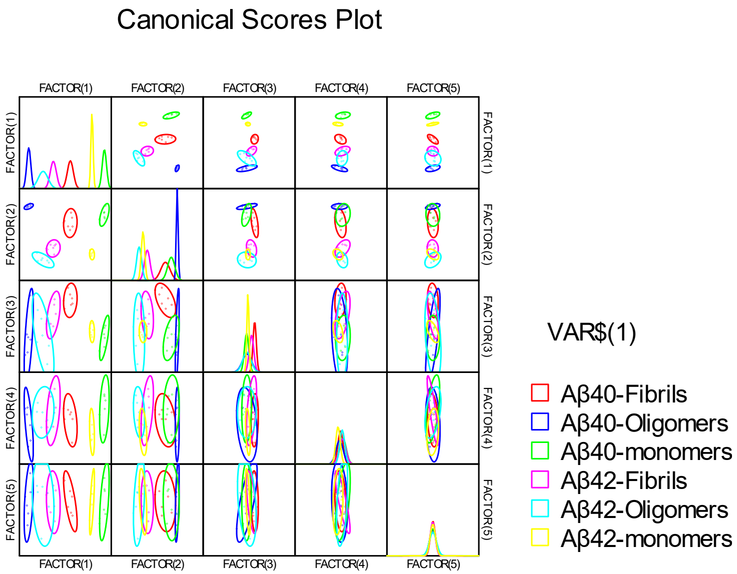

Supplementary Figure S6 Correlations of canonical fluorescence response patterns from an array of 6 channels screened by PCA against 1μM Amyloid- β peptides in serum. The 95% confidence ellipses for the individual analytes are shown.

Supplementary Table S11 Detection and identification of unknown samples in serum using LDA from the array of 6 channels screened by PCA. According to the verification, 22 among 24 unknown samples were correctly identified, representing an accuracy of 91.7%.

Analyte	Fluorescence response pattern						Results LDA					
Unknown	channel 1	channel 2	channel 4	channel 5	channel 6	channel 7	Factor 1	Factor 2	Factor 3	Group	Identification	Verification

samples												
1	0.13	0.07	0.17	0.07	0.06	0.04	6.12	4.37	-1.00	1	Aβ40-Fibrils	Aβ40-monomers
2	0.17	0.07	0.18	0.11	-0.05	-0.06	10.09	5.22	2.05	3	Aβ40-monomers	Aβ40-monomers
3	0.12	0.05	-0.19	0.08	-0.08	-0.07	15.08	5.36	0.19	3	Aβ40-monomers	Aβ40-monomers
4	0.18	0.10	-0.26	0.13	0.09	0.03	12.57	9.12	-3.25	3	Aβ40-monomers	Aβ40-monomers
5	0.05	0.00	0.12	0.04	0.17	0.60	-10.03	4.10	0.16	2	Aβ40-Oligomers	Aβ40-Oligomers
6	0.03	0.00	0.24	0.06	0.23	0.56	-12.26	4.03	-0.52	2	Aβ40-Oligomers	Aβ40-Oligomers
7	0.06	0.06	0.31	0.09	0.22	0.55	-11.95	3.94	-0.68	2	Aβ40-Oligomers	Aβ40-Oligomers
8	0.07	0.01	0.26	0.07	0.15	0.50	-9.48	4.25	1.39	2	Aβ40-Oligomers	Aβ40-Oligomers
9	-0.05	-0.07	-0.01	-0.07	0.14	0.05	4.28	0.93	-3.66	1	Aβ40-Fibrils	Aβ40-Fibrils
10	-0.06	-0.06	-0.03	-0.05	0.13	0.20	0.85	0.94	-3.13	1	Aβ40-Fibrils	Aβ40-Fibrils
11	0.03	-0.06	0.13	-0.01	0.08	0.06	3.50	2.59	-0.21	1	Aβ40-Fibrils	Aβ40-Fibrils
12	0.11	-0.05	0.14	-0.02	0.09	0.09	4.05	4.22	-0.22	1	Aβ40-Fibrils	Aβ40-Fibrils
13	-0.18	-0.13	-0.09	-0.13	0.12	0.02	4.35	-2.40	-4.65	6	Aβ42-monomers	Aβ42-monomers
14	-0.23	-0.16	-0.02	-0.15	-0.05	0.01	4.82	-5.40	-0.61	6	Aβ42-monomers	Aβ42-monomers
15	-0.20	-0.13	-0.07	-0.13	0.02	-0.05	6.48	-3.96	-2.74	6	Aβ42-monomers	Aβ42-monomers
16	-0.09	-0.14	0.00	-0.14	0.00	-0.03	7.21	-1.86	-1.06	6	Aβ42-monomers	Aβ42-monomers
17	-0.20	-0.16	0.51	-0.25	-0.08	0.42	-10.57	-10.05	3.29	5	Aβ42-Oligomers	Aβ42-Oligomers
18	-0.24	-0.16	0.52	-0.16	-0.08	0.34	-10.54	-8.99	4.51	5	Aβ42-Oligomers	Aβ42-Oligomers
19	-0.18	-0.16	0.43	-0.11	-0.10	0.37	-9.70	-6.29	6.19	5	Aβ42-Oligomers	Aβ42-Oligomers
20	-0.14	-0.24	0.49	-0.09	-0.03	0.24	-9.03	-3.44	7.07	4	Aβ42-Fibrils	Aβ42-Oligomers
21	-0.18	-0.14	-0.06	-0.09	0.14	0.34	-4.95	-1.36	-2.10	4	Aβ42-Fibrils	Aβ42-Fibrils
22	-0.18	-0.06	0.10	-0.14	-0.05	0.25	-0.47	-5.97	-1.03	4	Aβ42-Fibrils	Aβ42-Fibrils
23	-0.18	-0.02	0.24	-0.07	-0.05	0.20	-1.82	-5.83	-0.03	4	Aβ42-Fibrils	Aβ42-Fibrils
24	-0.10	0.10	-0.09	-0.02	0.13	0.38	-1.25	-1.37	-6.55	4	Aβ42-Fibrils	Aβ42-Fibrils

Supplementary Table S12 Training matrix of fluorescence response pattern from an array of channel 1-channel 10 against 1μM Amyloid-β peptides in serum. LDA was carried out and resulted in 3 factors of the canonical scores.

Analyte	Fluorescence response pattern										Result LDA			
Amyloid- β peptides	channel 1	channel 2	channel 3	channel 4	channel 5	channel 6	channel 7	channel 8	channel 9	channel 10	Factor 1	Factor 2	Factor 3	Group
Aβ40-monomers	0.11	0.07	-0.03	-0.05	0.07	-0.07	-0.08	0.04	0.05	-0.01	16.12	3.99	-0.08	3
Aβ40-monomers	0.21	0.12	-0.03	0.06	0.14	-0.03	-0.12	0.06	0.05	0.09	15.76	6.33	-0.76	3
Aβ40-monomers	0.09	0.04	-0.05	-0.03	0.05	-0.03	-0.13	0.01	-0.02	0.04	14.68	3.76	-2.41	3
Aβ40-monomers	0.09	0.06	-0.14	0.11	0.06	0.03	-0.17	0.00	0.01	0.02	15.13	4.15	0.65	3
Aβ40-monomers	0.13	0.06	-0.03	0.04	0.09	0.00	-0.17	0.03	-0.04	0.00	14.73	5.25	-2.31	3
Aβ40-monomers	0.13	0.08	-0.07	-0.07	0.08	0.00	-0.09	0.02	0.02	0.01	15.70	5.45	-1.24	3
Aβ40-Oligomers	0.16	0.14	0.12	0.47	0.17	0.32	0.49	0.00	0.02	0.04	-14.89	7.49	-1.99	2
Aβ40-Oligomers	0.15	0.10	0.11	0.43	0.14	0.28	0.54	-0.02	-0.02	0.03	-15.27	7.20	-1.54	2
Aβ40-Oligomers	0.14	0.07	0.12	0.47	0.13	0.31	0.46	-0.04	0.05	0.04	-14.67	6.73	-1.87	2
Aβ40-Oligomers	0.17	0.07	0.13	0.49	0.09	0.35	0.51	-0.07	0.04	0.01	-16.03	7.27	-2.68	2
Aβ40-Oligomers	0.12	0.08	0.08	0.35	0.14	0.29	0.50	0.01	0.04	0.04	-13.38	6.87	-1.04	2
Aβ40-Oligomers	0.23	0.02	0.12	0.48	0.06	0.22	0.55	-0.02	-0.07	-0.04	-14.09	7.89	-0.68	2
Aβ40-Fibrils	0.15	0.07	-0.03	0.24	0.11	0.04	0.17	0.05	0.04	0.04	3.91	5.13	2.43	1
Aβ40-Fibrils	0.04	0.02	-0.09	0.21	0.05	-0.01	0.12	0.01	0.02	0.06	4.78	1.98	3.23	1
Aβ40-Fibrils	0.06	0.02	-0.06	0.22	0.04	-0.04	0.20	0.00	0.01	0.00	3.69	2.31	4.30	1
Aβ40-Fibrils	0.09	0.02	-0.10	0.16	0.08	0.01	0.16	-0.01	-0.01	0.02	5.22	4.53	3.54	1
Aβ40-Fibrils	0.03	-0.03	-0.05	0.18	-0.01	-0.04	0.18	0.00	0.05	0.03	3.39	0.69	2.91	1
Aβ40-Fibrils	0.11	-0.03	-0.08	0.27	-0.02	0.07	0.20	0.04	0.00	-0.04	1.81	3.24	3.19	1
Aβ42-monomers	-0.18	-0.04	0.01	0.02	-0.03	-0.10	-0.11	-0.04	0.05	0.05	8.56	-4.49	-2.31	6
Aβ42-monomers	-0.17	-0.12	-0.06	0.00	-0.12	-0.09	-0.13	0.01	0.00	0.00	9.64	-5.29	-1.90	6
Aβ42-monomers	-0.20	-0.10	-0.08	-0.01	-0.11	-0.11	-0.09	-0.01	-0.05	0.04	8.28	-5.78	-2.10	6

Aβ42-monomers	-0.19	-0.13	-0.12	0.04	-0.13	-0.11	-0.14	-0.02	-0.02	0.02	9.89	-5.77	-0.54	6
Aβ42-monomers	-0.18	-0.12	-0.04	-0.02	-0.13	-0.08	-0.11	0.00	0.06	0.02	9.15	-5.80	-2.28	6
Aβ42-monomers	-0.18	-0.14	-0.06	0.03	-0.15	-0.07	-0.15	0.00	0.02	0.00	8.95	-6.03	-2.14	6
Aβ42-Oligomers	-0.24	-0.08	0.02	0.34	-0.08	0.03	0.27	-0.01	-0.01	0.06	-10.33	-6.65	-0.52	5
Aβ42-Oligomers	-0.22	-0.13	-0.06	0.28	-0.15	0.01	0.39	-0.03	-0.01	0.06	-10.69	-6.70	1.70	5
Aβ42-Oligomers	-0.25	-0.14	-0.01	0.24	-0.17	0.13	0.20	-0.04	0.01	-0.02	-8.35	-6.78	-2.85	5
Aβ42-Oligomers	-0.21	-0.15	-0.07	0.28	-0.17	0.08	0.30	-0.08	0.00	0.03	-9.14	-5.78	-0.06	5
Aβ42-Oligomers	-0.24	-0.12	-0.03	0.27	-0.16	0.06	0.22	-0.06	-0.02	0.01	-7.20	-6.76	-1.30	5
Aβ42-Oligomers	-0.18	-0.11	-0.04	0.29	-0.13	0.13	0.36	-0.05	0.03	-0.04	-10.02	-4.30	1.02	5
Aβ42-Fibrils	-0.17	-0.08	-0.06	0.14	-0.06	0.03	0.27	-0.02	0.02	0.03	-4.23	-3.47	0.86	4
Aβ42-Fibrils	-0.09	-0.05	-0.02	0.32	-0.07	0.06	0.26	0.03	0.11	0.02	-4.32	-3.29	2.49	4
Aβ42-Fibrils	-0.07	-0.04	0.00	0.33	-0.06	-0.02	0.25	-0.02	-0.01	0.03	-3.85	-2.76	1.53	4
Aβ42-Fibrils	-0.08	-0.09	-0.07	0.26	-0.10	0.02	0.24	-0.04	-0.03	0.03	-3.38	-2.34	1.12	4
Aβ42-Fibrils	-0.15	-0.10	-0.06	0.24	-0.09	0.04	0.29	-0.06	-0.02	0.01	-6.30	-3.38	1.18	4
Aβ42-Fibrils	-0.15	-0.12	-0.07	0.29	-0.13	-0.02	0.22	-0.03	-0.01	-0.01	-3.25	-4.89	2.46	4

Supplementary Table S13 LDA jackknifed classification matrix table obtained from the array of channel 1-channel 10 against 1μM Amyloid-β peptides in serum. The jackknifed classification matrix with cross-validation reveals a 100% accuracy.

	Aβ40-Fibrils	Aβ40-Oligomers	Aβ40-monomers	Aβ42-Fibrils	Aβ42-Oligomers	Aβ42-monomers	%Correct
Aβ40-Fibrils	6	0	0	0	0	0	100
Aβ40-Oligomers	0	6	0	0	0	0	100
Aβ40-monomers	0	0	6	0	0	0	100
Aβ42-Fibrils	0	0	0	6	0	0	100

Aβ42-Oligomers	0	0	0	0	6	0	100
Aβ42-monomers	0	0	0	0	0	6	100
Total	6	6	6	6	6	6	100

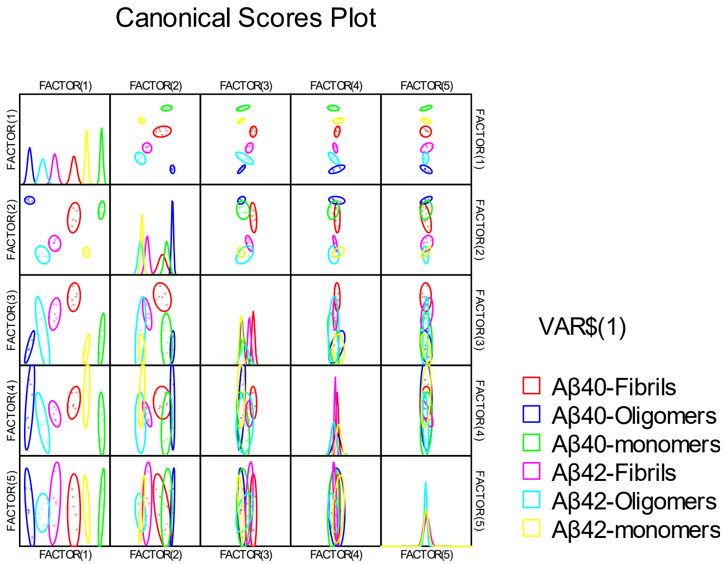

Supplementary Figure S7 Correlations of canonical fluorescence response patterns from an array of 10 channels against 1μM Amyloid-β peptides in serum. The 95% confidence ellipses for the individual analytes are shown.

Supplementary Table S14 Detection and identification of unknown samples in serum using LDA from the array of channel 1-channel 10. According to the verification, 20 among 24 unknown samples were correctly identified, representing an accuracy of 83%.

Analyte	Fluorescence response pattern	Results LDA

Unknown samples	channel 1	channel 2	channel 3	channel 4	channel 5	channel 6	channel 7	channel 8	channel 9	channel 10	Factor 1	Factor 2	Factor 3	Group	Identification	Verification
1	0.13	0.07	-0.14	0.17	0.07	0.06	0.04	0.07	0.03	0.05	9.04	4.61	2.53	1	Aβ40-Fibrils	Aβ40-monomers
2	0.17	0.07	-0.16	0.18	0.11	-0.05	-0.06	-0.01	0.04	0.02	15.73	6.05	5.77	3	Aβ40-monomers	Aβ40-monomers
3	0.12	0.05	-0.19	-0.19	0.08	-0.08	-0.07	0.01	-0.02	0.00	19.65	6.14	2.46	3	Aβ40-monomers	Aβ40-monomers
4	0.18	0.10	-0.15	-0.26	0.13	0.09	0.03	0.03	-0.01	0.01	15.24	9.80	-1.42	3	Aβ40-monomers	Aβ40-monomers
5	0.05	0.00	0.13	0.12	0.04	0.17	0.60	-0.04	-0.03	-0.03	-13.34	4.37	-3.02	2	Aβ40-Oligomers	Aβ40-Oligomers
6	0.03	0.00	0.02	0.24	0.06	0.23	0.56	-0.05	0.00	0.00	-13.74	4.72	-0.12	2	Aβ40-Oligomers	Aβ40-Oligomers
7	0.06	0.06	0.03	0.31	0.09	0.22	0.55	0.00	0.03	0.00	-12.60	4.60	1.19	2	Aβ40-Oligomers	Aβ40-Oligomers
8	0.07	0.01	0.12	0.26	0.07	0.15	0.50	-0.03	0.02	-0.02	-11.46	4.28	-0.94	2	Aβ40-Oligomers	Aβ40-Oligomers
9	-0.05	-0.07	-0.05	-0.01	-0.07	0.14	0.05	-0.03	-0.04	0.00	2.49	0.80	-5.29	1	Aβ40-Fibrils	Aβ40-Fibrils
10	-0.06	-0.06	-0.14	-0.03	-0.05	0.13	0.20	-0.03	-0.09	0.03	-0.19	1.66	-2.75	1	Aβ40-Fibrils	Aβ40-Fibrils
11	0.03	-0.06	-0.17	0.13	-0.01	0.08	0.06	-0.03	-0.01	0.01	5.81	2.99	2.05	1	Aβ40-Fibrils	Aβ40-Fibrils
12	0.11	-0.05	-0.12	0.14	-0.02	0.09	0.09	0.00	-0.06	0.01	4.81	4.52	-0.13	1	Aβ40-Fibrils	Aβ40-Fibrils

13	-0.18	-0.13	-0.15	-0.09	-0.13	0.12	0.02	-0.03	-0.08	-0.04	3.57	-2.02	-4.11	6	Aβ42-monomers	Aβ42-monomers
14	-0.23	-0.16	-0.16	-0.02	-0.15	-0.05	0.01	0.01	0.02	-0.05	6.64	-5.83	1.73	6	Aβ42-monomers	Aβ42-monomers
15	-0.20	-0.13	-0.15	-0.07	-0.13	0.02	-0.05	-0.03	0.06	0.02	7.44	-4.50	-1.00	6	Aβ42-monomers	Aβ42-monomers
16	-0.09	-0.14	-0.04	0.00	-0.14	0.00	-0.03	-0.02	-0.07	-0.09	6.48	-2.03	-3.57	6	Aβ42-monomers	Aβ42-monomers
17	-0.20	-0.16	-0.09	0.51	-0.25	-0.08	0.42	-0.07	-0.02	0.05	-11.02	-9.54	5.32	5	Aβ42-Oligomers	Aβ42-Oligomers
18	-0.24	-0.16	0.00	0.52	-0.16	-0.08	0.34	-0.10	-0.02	-0.01	-10.99	-8.52	4.24	5	Aβ42-Oligomers	Aβ42-Oligomers
19	-0.18	-0.16	-0.04	0.43	-0.11	-0.10	0.37	-0.04	-0.02	-0.03	-8.81	-6.13	6.55	4	Aβ42-Fibrils	Aβ42-Oligomers
20	-0.14	-0.24	-0.01	0.49	-0.09	-0.03	0.24	-0.02	0.04	-0.05	-8.25	-4.63	5.83	4	Aβ42-Fibrils	Aβ42-Oligomers
21	-0.18	-0.14	-0.07	-0.06	-0.09	0.14	0.34	-0.08	-0.05	0.05	-8.12	-1.35	-4.09	5	Aβ42-Oligomers	Aβ42-Fibrils
22	-0.18	-0.06	-0.07	0.10	-0.14	-0.05	0.25	-0.08	-0.03	-0.04	0.04	-4.78	0.79	4	Aβ42-Fibrils	Aβ42-Fibrils
23	-0.18	-0.02	0.00	0.24	-0.07	-0.05	0.20	-0.04	-0.06	-0.01	-2.50	-4.88	-0.19	4	Aβ42-Fibrils	Aβ42-Fibrils
24	-0.10	0.10	0.01	-0.09	-0.02	0.13	0.38	-0.04	0.01	0.01	-3.20	-0.15	-4.91	4	Aβ42-Fibrils	Aβ42-Fibrils
